# Broadening the Dental Hygiene Students’ Perspectives on the Oral Health Professionals: A Text Mining Analysis

**DOI:** 10.3390/dj10090160

**Published:** 2022-08-29

**Authors:** Yukiko Nagatani, Rintaro Imafuku, Yukie Nakai

**Affiliations:** 1 Department of Dental Hygiene, University of Shizuoka Junior College, 2-2-1 Oshika, Suruga-ku, Shizuoka-shi 422-8021, Japan; 2 Medical Education Development Center, Gifu University, 1-1 Yanagido, Gifu 501-1194, Japan

**Keywords:** professional identity formation, dental hygiene student, perceptions of professional, health professions education, undergraduate education, communities of practice

## Abstract

Professional identity formation, an important component of education, is influenced by participation, social relationships, and culture in communities of practice. As a preliminary investigation of dental hygienists’ professional identity formation, this study examined changes in the dental hygiene students’ perceptions of oral health professionals over the three years of their undergraduate program. At a Japanese dental hygiene school, 40 students participated in surveys with open-ended questions about professional groups several times during their studies. The text data were analyzed through content analysis with text mining software. The themes that characterized their dental hygienist profession perceptions in their programs each year were identified as: “Supporters at the dental clinic”; “Engagement with interprofessional care” and “Improved problem-solving skills for clinical issues regarding the oral region”; and “Active contribution to general health” and “Recognition of the roles considering relationships” (in the first, second, and third years, respectively). The students acquired professional knowledge and recognized the significance and roles of oral health professionals in practice. They gained more learning experiences in their education, including clinical placements and interprofessional education. This study provides insight into curriculum development for professional identity formation in dental hygiene students.

## 1. Introduction

Dental hygienists specialize in the control of dental diseases; this profession is prevalent and generally professionally licensed in over 30 countries [1,2]. They are primarily responsible for preventive procedures and non-surgical periodontal treatments. Their role is usually to investigate dental diseases, and motivate and instruct patients about dental health [1,2,3]. In Japan’s aging society, the necessity of implementing oral health management as primary care is becoming widely recognized by health professionals and the public. It is essential for dental hygienists to prevent and treat dental diseases and support general health by maintaining and promoting the oral environment [4], and improve the quality of life [5]. To respond to these social needs, it is necessary to secede from the emphasis on manual procedures in dental clinics [6] and practice as directed by the dentists; to train the dental hygienists with high qualities and professional skills who can think and practice on their own in the community in cooperation with multiple professionals; and to establish a way of the dental hygiene work that is adapted to the aging society [7]. Therefore, it is necessary to develop a deep understanding of one’s professional role and expertise, in addition to the roles of the other professionals with whom they work, considering social needs. Moreover, the internalization of this value system promotes the identity formation of a learner who practices in the health profession.

Identity formation is “the process by which an individual self-defines as a member of that profession based on the acquisition of the requisite knowledge, skills, attitudes, values and behaviors” [8]. It is considered an important component of education in the health profession. In identity formation in health professions, social interactions, experiences, and learning contexts are influential factors. At the collective level, it is influenced by context, including culture and the learning environment chosen by the learner. According to Wenger, gaining practical experience through active engagement and interactions with other members of a community is essential to professional identity formation [9].

In health profession education, the learners’ profound transformation occurs through learning experiences, most notably the clinical ones [10,11,12]. To make the transition from a layperson to a professional, they need learning opportunities that encourage a re-identification of the past (or the present) self [13]. Furthermore, building social relationships with peers and mentors in clinical education, as well as reflecting and discussing about learning experiences in clinical settings, strengthens professional identity formation through socialization with professional groups [14,15]. Therefore, the health profession education programs need to be structured stepwise from the first year to enable the development of values and professional identity regarding evidence-based practice and professionalism [16].

The importance of introducing education and support that promotes professional identity formation in dental hygiene education has been internationally recognized. For example, service-learning exercises and curriculum revisions have been implemented to develop students’ attitudes and sense of professional responsibility [17,18]. In addition, Imafuku et al. explored the interprofessional identity formation in dental hygienists [19]. However, the overall understanding of the process of dental hygienists’ professional identity formation over time, beginning with their first year of study, remains unclear. Clarifying the process of dental hygienists’ professional identity formation could provide insight into students’ actual perceptions of the nature, tasks, and value system of the profession. Analyzing the discrepancy between their perceptions of the profession of dental hygienist and the learning outcomes expected by their teachers would provide a basis for improving and developing new educational strategies and learning support methods in the future. Therefore, in this study, as a preliminary investigation of dental hygienists’ professional identity formation, we examined the changes in their perceptions of the dental hygienist profession during the three years of their undergraduate education.

## 2. Materials and Methods

### 2.1. Participants

An observational, longitudinal, and prospective study was conducted in the Department of Dental Hygiene at the University of Shizuoka Junior College. All freshmen in the cohort who enrolled in the school in 2019 were invited to participate in this study, and all consented (*n* = 40). They were 18–19 years old at the time of enrollment. Their background prior to enrollment was high school or post-high school graduates preparing for university entrance exams. In addition, none of them had clinical experience in the medical field.

### 2.2. Data Collection

An open-ended questionnaire was self-administered to 40 students each year for three years, from the second half of their first year to the second half of their third year. The open-ended format allowed the researchers to elicit richer data on students’ perceptions of the professional roles and prospects as health professionals at each stage of their undergraduate education. Specifically, this questionnaire survey was structured by five open-ended questions about their reasons for choosing the profession, their ideal future image as a dental professional, their perceptions of professionalism in dental hygiene, societal expectations, and competencies required for the profession. The actual questions in the survey are as listed below:

- Why did you decide to become a dental hygienist?
- What kind of dental hygienist do you want to be?
- What do you think is the professionalism of dental hygienists?
- What do you think is required of you as dental health professionals by society and patients?
- What competencies do you think that dental hygienists need to have for clinical practice?

The data were gathered in October 2019 (at the beginning of the first year’s second semester), October 2020 (at the beginning of the second year’s second semester), and November 2021 (at the end of the clinical practice).

### 2.3. Data Analysis

Data were transcribed from questionnaires and then subjected to text mining analysis using the software, KH Coder 3 (Koichi Higuchi, Kyoto, Japan) [20]. The software produced a list of words according to their frequencies and interrelationships. It is a quantitative process of adapting algorithms to discover hidden, useful, and interesting patterns in unstructured qualitative text data [21,22,23]. In analyzing the qualitative text data, text mining increases the reliability and validity of the coding [23]. In addition, it can be used in conjunction with human coding content analysis to enhance the rigor [24].

In this study, the software was used for the research participants’ perceptions of the dental hygienist profession to obtain an overall picture of the diversity, type, and distribution as a framework for understanding the data before conceptualization. A hierarchical cluster analysis was conducted on the pupils’ perceptions of the oral health professionals at each of the three data collection points; moreover, frequently occurring words were extracted. Subsequently, the co-occurrence of the strength of the association between the extracted words was calculated using the Jaccard coefficient. In these analyses, the Key Word in Content (KWIC) concordance function was used to identify words with three or more occurrences to qualitatively examine students’ interpretations of their perceptions of the dental hygienist and how they changed over time. The resulting associations were visually represented on a Co-Occurrence Network Map and coded and categorized by the first and second authors to increase rigor and explore the meaning of the data in conjunction with the qualitative analysis. The authors discussed and identified underlying themes in the visualization of the results. Preliminary results were then discussed and a consensus was reached by all members of the research team. The chi-square test was applied to assess the significance of differences in the distribution of students’ perceptions of oral health professionals at each of the three data collection points using KH Coder, version 3 (Koichi Higuchi, Kyoto, Japan). Statistical significance was set at *p* < 0.05.

In addition, the relationship between the results of each period and the content of the curriculum taken up to that point, as well as a comparison of the data at the end of the third-year field training with the diploma, were also examined.

### 2.4. Study Context

This study was conducted at the Department of Dental Hygiene, Junior College of the University of Shizuoka, involving students enrolled in a three-year education program. Regarding the educational philosophy, this school follows the diploma policy, which entails the student learning outcome objectives and graduation approval/degree awarding program; it is set to train dental hygienists who can respond to the oral health needs of the community. A junior college bachelor’s degree is awarded to those who have studied in an educational program to acquire the abilities listed below and have earned the necessary credits.

- Professional knowledge, abilities, and communication skills related to dental hygiene
- Logical thinking and problem-solving skills
- Awareness of their roles and responsibilities as dental hygiene practitioners and the ability to perform them appropriately
- A rich sense of humanity and high ethical standards, and ability to collaborate and cooperate with other professionals
- Contribution to the development of people’s health and a striving for lifelong learning.

**Figure 1:**
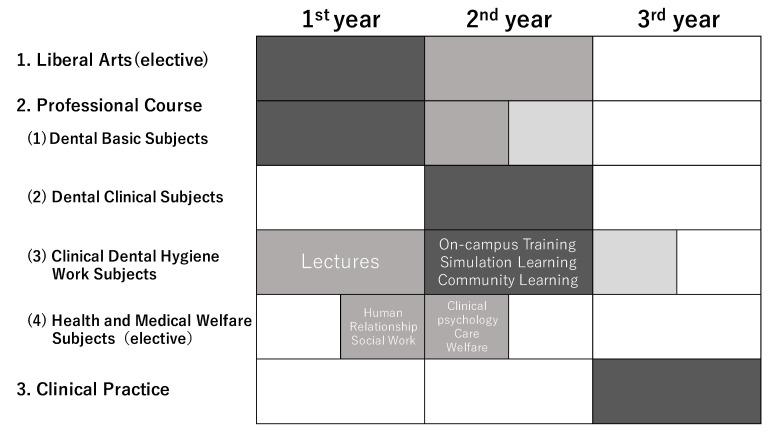
Three-year curriculum of dental hygiene at the research site. (The intensity of the color indicates the volume of the subject’s content.)

In the training school included in this study, the curriculum was designed to enable students to achieve the above-mentioned diploma. As shown in Figure 1, in the first year, students attend lectures on liberal arts, basic specialties, and dental hygiene. In the second year, they opt for specialized clinical subjects, on-campus training related to dental hygiene work, and subjects related to interpersonal support and well-being in collaboration with students from other departments. In addition, elective subjects related to health and medical well-being have been established from the second semester of the first year to the first semester of the second year. After studying these subjects, the third year includes off-campus practical training in dental clinics, oral surgery departments of general hospitals, nursing homes, and disability support facilities.

### 2.5. Ethical Considerations

This study was approved by the University of Shizuoka Research Ethics Committee [approval number 1-19]. Furthermore, informed consent was provided by all the study participants.

## 3. Results

### 3.1. Changes in the Word Frequency

Overall, 333 statements were related to the professional role and attitude of dental hygiene, of which 120, 177, and 192 statements were categorized for the first, second, and third years, respectively. The following five words were found to occur over 30 times in total:

- Oral: 90 times
- Patient: 64 times
- Dental: 62 times
- Health: 51 times
- Knowledge: 35 times

### 3.2. Number of Code Occurrences

**Figure 2:**
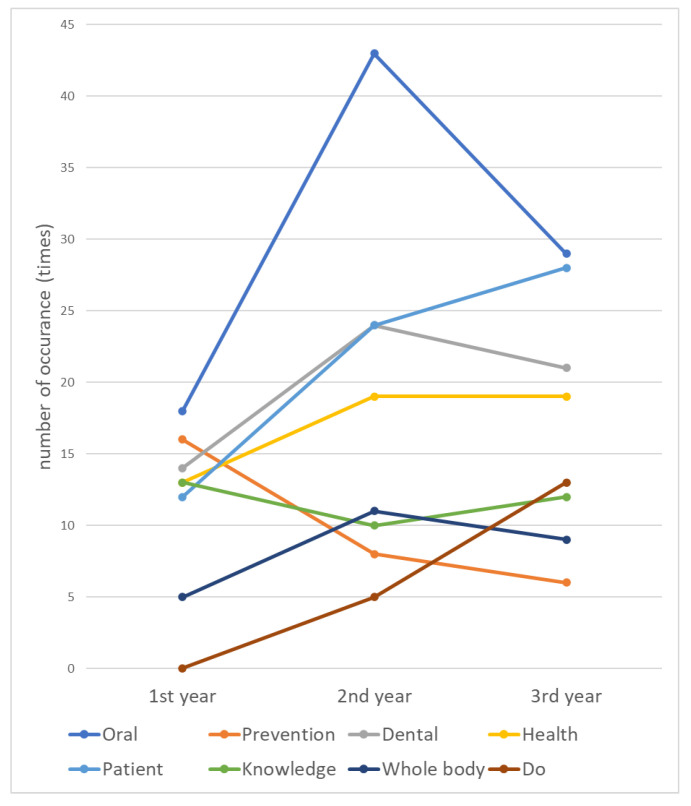
The number of the extracted words of expertise perceived by the dental hygiene students over time.

The occurrence rate of the aforementioned words varied from year to year (Figure 2). In the first year, “oral” was the most frequently appearing word, followed by “prevention”, “dental”, “health”, “knowledge”, and “patient”; in the second year, “oral” was the most frequently occurring word, similar to the first year, followed by “patient”, “dental”, “health”, “whole body”, and “knowledge”. The increased utilization of the words “patient” and “whole body” was characteristic. The former occurred 12, 24, and 28 times in the first, second, and third years of school, respectively, and the number of occurrences tended to increase as the school year progressed. In the third year, “oral” was the most frequently used word, followed by “patient”, “dental”, “health”, “do”, and “knowledge”, similar to the first and second years. Although “do” did not occur in the first year, it showed an increasing trend with 5 and 13 occurrences in the second and third years, respectively. However, the number of occurrences of “prevention” tended to decrease with increasing grade level, from 16 to 8 and 6 times in the first, second, and third years, respectively.

### 3.3. Code Occurrence Rate

**Figure 3:**
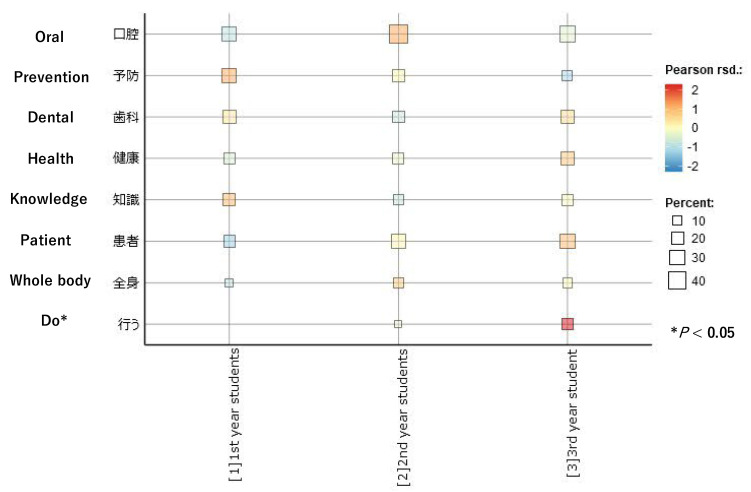
Changes in the occurrence of the specialty codes as perceived by the dental hygiene students.

Figure 3 visualized the changes in the occurrence rate of the specialty codes as perceived by the dental hygiene students over time. The square size indicates the occurrence rate of each code (showing “percent”), and its color corresponds to the standardized residuals (Pearson residuals). Therefore, a larger square represents a higher rate of occurrence, and the darker color represents the larger residual. According to Figure 3, only “do”, which did not appear in the first year, showed a significant difference in the proportion of the occurrence of the three points (*p* < 0.05, Chi-square test).

### 3.4. Recognition of Professional Roles, Competence, and Attitude at the Three Time Points Based on the Strength of the Relationship between the Words

From the co-occurrence network model diagram showing the strength of the association between the words with three or more occurrences, the characteristic themes were extracted for the dental hygiene students’ perceptions of their professional roles, competence, and attitude at each of the three time points, using the KWIC concordance function to assess the word usage.

#### 3.4.1. At the Beginning of the First Year’s Second Semester

**Figure 4:**
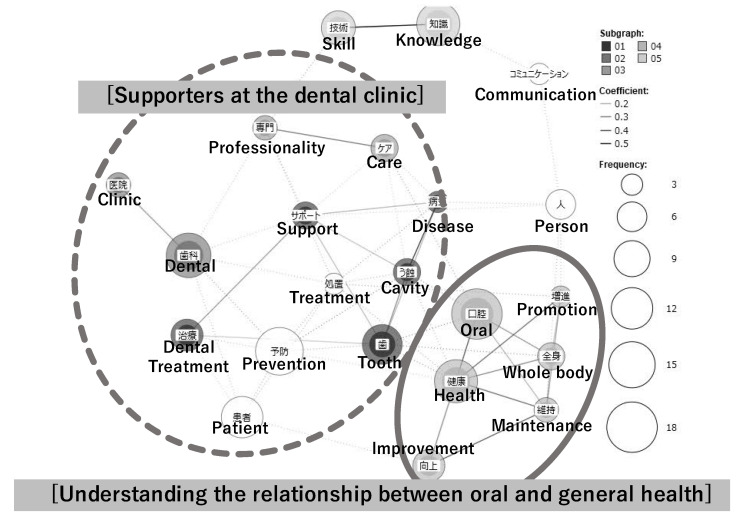
The co-occurrence network diagram of the first-year dental hygienists’ perceptions of professional role and competencies.

During the first year, the following two themes were extracted (Figure 4):

##### Supporters at the Dental Clinic

The dental hygienists were identified as assistants in the dental clinic, professionals who help patients improve their health by providing expert knowledge and skillful care in the prevention and treatment of dental caries, alleviating their fear of treatment, and supporting the dentists during the dental treatment.

- “How to comfort the patients at the dental clinic, a place where they are considered to be uncomfortable”.
- “Supporting the dental treatment to run smoothly”.

##### Understanding the Relationship between Oral and General Health

The participants were aware that the oral region and the whole body are deeply related; however, as professionals in oral health, they believed that they must enhance their knowledge, treatment, instruction, and care techniques for “improving the health of the whole body through oral care”.

- “Understanding better than anyone else that the teeth and oral health are linked to general health”.
- “Dental caries prevention and understanding the relationship between the oral region and the whole body”.
- “The ability to communicate the importance of oral health in various settings, both in the medical and educational fields”.

#### 3.4.2. At the Beginning of the Second Year’s Second Semester

**Figure 5:**
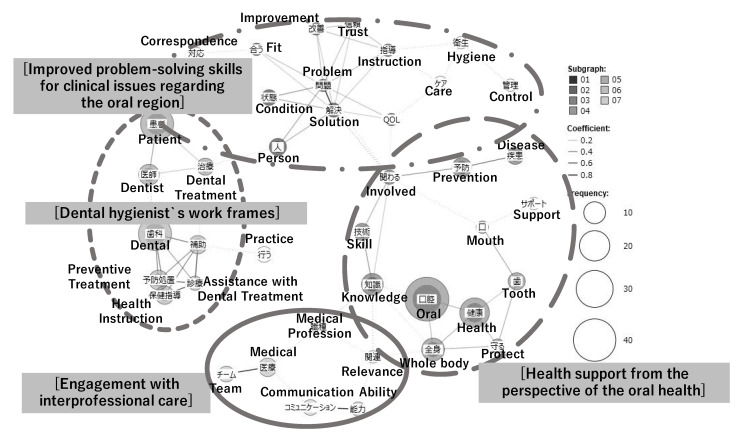
The co-occurrence network diagram of the second-year dental hygienists’ perceptions of professional role and competencies.

During the second year, the following four themes were extracted: (Figure 5).

##### Dental Hygienists’ Work Frames

Participants recognized the framework of the three main tasks of a dental hygienist in Japan, including caries prevention treatment through fluoride application, assistance work for smooth dental treatment by standing between patients and dentists, and oral health instruction.

- “Must be able to assist in the dental treatment, provide the oral health instruction, and perform the caries prevention procedures”.
- “A person who can stand between the dentist and the patient and contribute to enhanced treatment”.

##### Engagement with Interprofessional Care

The participants felt they must have a broad knowledge of medicine, not only regarding oral health, which is their profession, but also systemic diseases. They aimed to confidently collaborate and communicate with multiple professionals and develop the ability to handle interprofessional medicine that could provide patient-centered care.

- “To acquire an awareness of the whole body, not just the oral, and to be able to perform team medicine with other professionals”.
- “Have a patient-centered attitude and the communication skills to practice in a team-based environment”.

##### Health Support from the Perspective of the Oral Health

The need for dental hygienists to acquire medical information along with oral health knowledge in order to demonstrate support for general health from the perspective of oral health has been recognized. These skills help prevent infection and improve general health and quality of life by helping to maintain and promote oral health throughout life, including the perioperative period and old age.

- “To improve the quality of life as well as the oral health of patients”.
- “Promoting and maintaining general health through the oral health”.

##### Improved Problem-Solving Skills for Clinical Issues Regarding the Oral Region

The participants were aware of their role in solving and improving oral problems. Therefore, participants recognized the need for the ability to provide medical care, including instruction, care, and hygiene management, to identify and resolve issues based on each person’s condition status and needs, and to build a relationship of trust with the patient.

- “Perceive the patients’ condition, their oral health situation, understand their problems, and provide them with appropriate care to resolve those issues”.
- “I think it is about improving oral health, instructing about oral health, and building trusting relationships to solve oral health problems”.

#### 3.4.3. At the End of the Third-Year Clinical Practice

**Figure 6:**
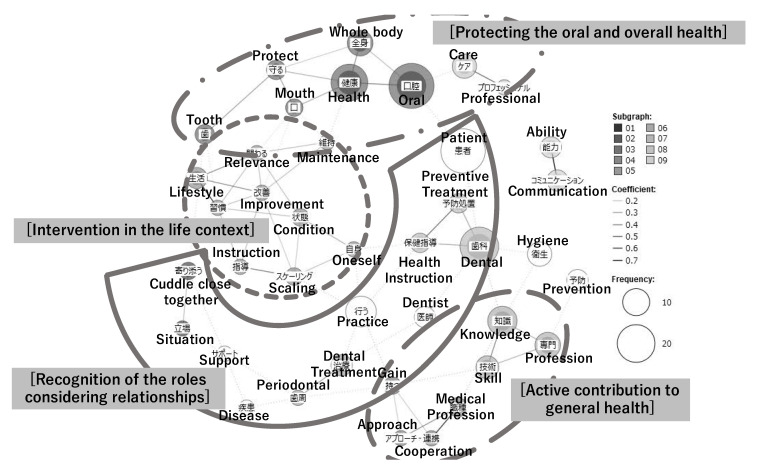
The co-occurrence network diagram of the dental hygienists’ perceptions of professional role and competencies in the third year.

At the end of the third-year clinical practice, the following four themes were identified: (Figure 6).

##### Active Contribution to General Health

Participants mentioned the need for evidence-based and correct professional knowledge and skills, as well as the ability to make decisions in clinical practice. They recognized that acquiring these competencies would enable them to respond to patient complaints and work smoothly with multiple health care professionals from an oral health perspective.

- “Dental as well as medical knowledge and working with various professionals to approach problems”.
- “As a dental hygienist, we must have the knowledge to answer patient complaints”.

##### Recognition of the Roles Considering Relationships

As professionals supporting patients’ autonomous oral health care, they recognized their role in improving self-care and providing professional care based on their relationship with patients; they were able to incorporate specific preventive approaches such as dietary instructions and periodontal treatments. In addition, they recognized their role based on their clinical relationship with the dentist, which included managing the patients overall health during treatment and paying attention to the intentions and feelings of both the patient and the dentist.

- “We are professionals in terms of prevention; thus, hygienists are required to provide care and prevention so that we do not get to the treatment stage”.
- “To support the patients’ feelings (chief complaint) and to help them feel comfortable through the dentist”.

##### Protecting the Oral and Overall Health

The students expressed their intention to be actively involved in the whole health management not limited to oral health care, such as supporting the daily quality of life from a holistic perspective.

- “Improvement of the overall health and quality of life through oral health”.
- “To enhance the quality of life by improving and maintaining oral health that is closely related to the everyday life, such as eating, speaking and laughing”.

##### Intervention in the Life Context

In the approach to oral health maintenance and promotion, participants recognized the need to not only understand the oral and general condition of patients, but also to actively incorporate their oral function and hygiene into their daily lives by intervening in their life background, such as their diet and lifestyle.

- “To be able to provide health instruction from a dental perspective, including dietary instruction and lifestyle modification”.
- “To provide instruction for the patients’ own oral health self-care, so that they themselves can maintain a good oral health”.

## 4. Discussion

This study found that dental hygiene students’ perceptions were strongly influenced and shaped by their learning experiences during college. This included lectures on their specific duties, the interdisciplinary education program, and clinical placements in dental facilities. As previous studies have shown, it is critical that they gain a clear understanding of the core values, expectations, and behaviors of the profession during health professions education and are encouraged to develop their own identities in addition to acquiring knowledge and skills [14,25]. Accordingly, the present longitudinal research demonstrated four aspects of changing the perceptions of the oral health professional among dental hygiene students.

The first aspect is the relationship between knowledge acquisition and professionalism. The establishment of a knowledge system in a professional domain has a significant impact on identity formation [26,27]. Moreover, self-efficacy has been related to professional recognition, skill mastery, and knowledge [28]. In this research, the first-year students were expected to acquire knowledge related to the “oral and general health promotion” such as “dental caries prevention and understanding the relationship between the oral region and the whole body” and “the teeth and oral health are linked to general health”. In the second year, the concept of the “dental hygienists’ work frames” learned in lectures such as “assist in the dental treatment, provide the oral health instruction, and perform the caries prevention procedures”, was often mentioned as a competence of dental hygienists. Specifically, in the first and second years, their professionality was frequently viewed from the aspect of knowledge acquisition reflected in the content of the specialized clinical courses. Knowledge acquisition is also essential in forming the professional identity foundation [26]. Nevertheless, in the first and second years, the students tended to focus excessively on obtaining the expertise of a dental hygienist. As it is said that identity formation is facilitated by active engagement and interaction in the community [9], therefore, education programs that promote the pupils’ understanding of “Recognition of the roles considering relationships”, such as communication training, interprofessional education, and early exposure program need to be developed and incorporated into the basic education stage. For example, early exposure is an educational strategy to help students adapt to the clinical environment, improve their skills of reflection and evaluation, and establish their professional identity [29,30,31].

Regarding knowledge acquisition, enhancing logical thinking and problem-solving skills are key for curriculum development. Specifically, it is important to incorporate research and exploratory activities into each subject area step-by-step in dental hygiene education. These skills are necessary to apply the acquired knowledge in practice. In medical education, undergraduate research courses in which pupils independently conduct research for a certain time have been offered; further, efforts to improve their logical thinking and evidence-based medicine have been reported [32,33]. Similarly, in the three years of dental hygiene education, it is necessary to promote a continuum of learning through exploratory activities about assessment, planning, implementation, and evaluation to develop logical thinking and problem-solving skills for evidence-based medical practice [34]. Therefore, educational objectives and content should be jointly determined by the clinical practice organization and the educational institution, and research activities should be integrated into the curriculum.

The second aspect is the students’ broadened perceptions of their professional roles and responsibilities, which influence professional identity formation [35,36,37]. Thus, this study indicated the changes in the pupils’ perceptions of the role and responsibilities of a dental hygienist. Specifically, the first-year students limited the place of work to the “dental clinic” and viewed dental hygienists as professionals involved in the dental care there; further, there was a limited term or phrase indicating a professional role of caring for people at different life stages, such as oral function management in the elderly and perioperative oral care. In the second year, the pupils acquired the perspective of the “patient” and the theme of supporting patient health from the oral health perspective was extracted. In the third year, the theme of protecting the patient’s overall health as a role of a dental hygienist was obtained. These results suggested that health is not limited to the oral region but is also considered from the standpoint of the whole body and that the students had been taking more initiative in “protecting” the patient’s health. Furthermore, the vague image of the role of a dental hygienist in the first year was transformed into a more concrete image and gained a more generalist view. They were able to think about the clinical issues from a patient’s standpoint in the third year. Their text data included “improving and maintaining oral health that is closely related to everyday life, such as eating, speaking and laughing”, and the “improvement of the overall health and quality of life through oral health”. This result is consistent with that of the study by Imafuku et al., that explored the identity formation processes of dental hygienists in the field as interprofessional collaborators [19]; however, the significance of this research is that it showed that such a change in perception might be occurring among the undergraduate students.

In dental hygienist education, for example, it is essential to provide opportunities for the students to reaffirm professional values and beliefs, including what kind of a dental hygienist they want to become, and to clarify their vision for the future in their education’s early stage. In clinical education, dental hygienists and other professionals can assume specific roles within the community of practice through collaborative practice. To encourage group-level socialization, opportunities to meet role models who are active in various situations [8,38] and internships in a community of practice can be used. It is also important to incorporate into the curriculum supportive methods that internalize the values of the dental hygienist during these social experiences [8,25,38,39,40]

The third aspect is a change in perception from individual to team care. Over three years, this study’s participants modified their perceptions of the dental hygienist’s professional role and responsibilities from a focus on the “individual” as a specialist involved in the dental treatment to an “interpersonal” or “team” perspective, such as interprofessional collaboration and patient communication. In the first year, many of the texts referred to their skills, such as “dental caries prevention and understanding the relationship between the oral region and the whole body” and “the ability to communicate the importance of oral health in various settings, both in the medical and educational fields”. Similarly, in the second year, there were several statements about their skills; however, the concepts related to the professions in which they work with the people they care for, such as “provide them with appropriate care to resolve these issues” and “have a patient-centered attitude and the communication skills to practice in a team-based environment”, were more common. During this time, students are believed to have gained a deeper understanding of the relationship between oral and general health, as well as a perspective on the importance of the dental hygienist’s intervention to a person’s general health through the study of specialized clinical subjects. In addition, learning about welfare, management, and clinical psychology, planning dental health instruction, and experiencing being in the role of a patient during campus clinical training may have fostered an awareness of the need for interprofessional collaboration, the ability to provide interpersonal support, and the responsibility to solve health problems. In the third year, students interacted with patients and dental hygienists in clinical practice. They noted the relationships between patients and medical staff, patients and dental hygienists, and dental hygienists and various professionals. Students mentioned the need for clarification of the relationship and their own roles between dental hygienists and multidisciplinary professionals, the ability to respond to patient complaints, the ability to make judgments that allow active participation in multidisciplinary care confidently, and scientifically sound clinical skills.

This finding corroborates the results of previous studies, which show that students focus more on enhancing teamwork and collaborative practice, and their identity confidence is increased after interprofessional education programs [41,42]. However, this study did not confirm the pupils’ improved collective learning skills, such as consensus-building and leadership, and the relationships between their interprofessional learning experience and quality care provision, as suggested by Cooper and Imafuku [41,42]. A possible factor influencing this is that during their clinical practice at the university, the pupils themselves had limited opportunities to experience interprofessional collaborative practice with professionals other than dentists.

Regarding interprofessional collaboration, most clinical practice hours were spent in private dental clinics, presumably because there were not enough opportunities to experience situations of interprofessional collaboration with other professionals. During the five-day oral surgery clinical practicum in the general hospital, students did not proactively practice interprofessional collaboration due to the severity of patients’ illnesses, systemic management issues, and limited skills; it is assumed that they only observed the instructor and had difficulty recognizing interprofessional collaboration. Furthermore, there is a strong perception that they are employees who perform their duties under the instruction of the Dental Hygienists Act, which states that “dental hygienists are those whose work is to perform the following acts under the instruction of dentists and preventive measures for diseases of the teeth and oral” [43]. Annan-Coultas indicated that observations of real-life teamwork environments can be a meaningful way to teach interprofessional teamwork concepts [44]. Similarly, although it is difficult for pupils to practice interprofessional teamwork on high-risk patients in a clinical setting, it may be useful for them to observe a multidisciplinary conference and how dental hygienists practice collaborative medicine in such a situation to deepen their awareness of interprofessional collaboration.

Finally, analysis of the textual data suggests that dental hygiene students have a stronger sense of ownership as health care providers. It suggests that participation in the clinical placements strengthened students’ professional identity and sense of responsibility. Specifically, text analysis in the third year showed that the verbs “to do” and “to protect”, which indicate proactive action in terms of professional role and responsibility, were frequently described. This change may be primarily due to their insertion into the dental clinic community during their field training and gaining experience in clinical practice, albeit in a peripheral role, while building relationships with the dentists and dental hygienists in the dental clinic. Students’ identities are formed not only by their associations with professional networks, but also by their actual experiences and participation in the community [9].

The increased sense of ownership is related to student’s attitude toward lifelong learning. Thus, further educational support is needed from the first-year education to continuously enhance their awareness of the importance of self-learning in healthcare. Lifelong learning is an integral part of the communities of practice, with time and expectations for reflection [45]. Encouraging pupils to reflect on their experiences in clinical placements deeply can enhance their awareness of lifelong learning and cultivate their self-learning skills. Some studies in medical education show that medical students’ identities are promoted in relationships through reflective activities and the need to provide educational spaces for them to understand their identity as a doctor by recounting their clinical experiences [14,46,47].

In this study, we conducted a content analysis of an open-ended questionnaire related to the role and competencies of oral health professionals in dental hygiene students. The results of this study have implications for society as a whole, particularly in the areas of dental education and health care. However, the questionnaire survey did not reflect the background of the students’ descriptions, what they could not express, and the depth of their insight. In particular, we did not explore the kind of individuality and specific experiences from which their perceptions emerged, how they perceived their experiences, and what kind of identity they formed in the narrative process. In addition, although we related the changes in students’ perceptions to the curriculum, we were unable to examine the influence of extracurricular factors, such as extracurricular activities and prior learning experiences. As the data in this study were compiled and compared across all participants at the three time points, changes in individual perceptions over time could not be tracked and the effects of individual personalities and environments could not be analyzed. Therefore, further qualitative interview studies are needed to more fully incorporate students’ experiences behind their perceptions of professionals and to shed light on professional identity formation among dental hygienists.

## 5. Conclusions

The dental hygiene students in this study acquired professional knowledge, became aware of the importance and role of oral health professionals in practice, and broadened their perspectives as dental hygienists through their health care education, which included lectures, clinical placements, and interprofessional education. Therefore, this study provides insight into the development of curricula to promote the professional identity of dental hygiene students.

## Data Availability

The data presented in this study are available on request from the corresponding author.
